# Causal associations between genetically determined common psychiatric disorders and the risk of falls: evidence from Mendelian randomization

**DOI:** 10.1186/s40001-023-01502-y

**Published:** 2023-12-09

**Authors:** Haitao Zhang, Chuanglong Xu, Chunchun Yuan, Binhao Shi, Wenhao Zhu, Hongyu Wang, Furui Fu, Dezhi Tang, Yongjun Wang

**Affiliations:** 1 Longhua Hospital, Shanghai University of Traditional Chinese Medicine, Wan-Ping South Road 725#, Xuhui District, Shanghai, 200032 China; 2 Spine Institute, Shanghai Academy of Traditional Chinese Medicine, Shanghai, China; 3 Key Laboratory of Theory and Therapy of Muscles and Bones, Ministry of Education, Shanghai, China; 4 Ningxia Hospital of Traditional Chinese Medicine and Chinese Medicine Research Institute, Ningxia, China; 5 Shanghai University of Traditional Chinese Medicine, Shanghai, China; 6 Shanghai Municipal Hospital of Traditional Chinese Medicine, Shanghai University of Traditional Chinese Medicine, Shanghai, China

**Keywords:** Schizophrenia, Major depressive disorder, Alzheimer’s disease, Falls, Causal associations, Mendelian randomization

## Abstract

**Background:**

The causal associations between psychiatric disorders and falls risk remains uncertain. Consequently, this study aimed to explore the causal relationship between genetically determined three common psychiatric disorders and the risk of falls based on Mendelian randomization (MR).

**Methods:**

The genome-wide association study (GWAS) data for schizophrenia (SCZ) (*N* = 320,404), major depressive disorder (MDD) (*N* = 480,359), and Alzheimer's disease (AD) (*N* = 63,926) were obtained as exposures. The GWAS data for falls risk (*N* = 451,179) was obtained as outcome. Univariate Mendelian randomization (UVMR) was used to evaluate the direct causal relationship between SCZ, MDD, AD, and risk of falls. Inverse variance weighting (IVW) was used as the primary analysis method. Sensitivity analysis was performed to assess the validity of the casualty. Multivariate Mendelian randomization (MVMR) analysis was conducted after adjusting body mass index and smoking initiation. Mediating MR was conducted to calculate the mediating effects of potential intermediaries.

**Results:**

UVMR analysis showed that SCZ (OR 1.02, 95% CI 1.01–1.04, *p* = 8.03E−03) and MDD (OR 1.15, 95% CI 1.08–1.22, *p* = 1.38E−05) were positively associated with the risk of falls. Sensitivity analysis results were reliable and robust. MVMR results indicated that the relationship between MDD and SCZ and falls risk remained significant. Mediating MR results demonstrated that smoking initiation mediated partial causal effect of SCZ (0.65%, *P* = 0.03) and MDD (14.82%, *P* = 2.02E−03) on risk of falls.

**Conclusions:**

This study provides genetic evidence for a causal relationship of individuals with SCZ and MDD on an increased risk of falls. Healthcare providers should be aware of the risk of falls in MDD and SCZ patients and develop strategies accordingly.

**Supplementary Information:**

The online version contains supplementary material available at 10.1186/s40001-023-01502-y.

## Introduction

Falls represent an escalating global public health concern and now rank as the second most prevalent cause of unintentional injury-related fatalities, trailing only traffic accidents [1]. The World Health Organization has reported that approximately 684,000 individuals worldwide die as a result of falls annually, with low- and middle-income countries accounting for more than 80% [2]. The impact of falls on individuals' daily lives and mental health can be severe and far-reaching, with consequences ranging from fractures and brain injury to chronic pain, loss of independence, fear of falls, disability, and even death [3]. Simultaneously, falls and related injuries put a substantial economic burden on patients and the national health department [4, 5]. According to a systematic review, fall-related costs make up approximately 0.85–1.5% of total medical expenses in Europe, North America, and Australia [6]. Hence, it is urgent to gain a comprehensive understanding of the high-risk falls population to implement effective interventions.

Falls commonly caused severe injuries among older adults with mental disorders [7]. Elderly patients in psychiatric hospitals were at higher risk of falls compared to general hospitals [8]. Mental disorders predominantly manifest as chronic and complex conditions involving abnormal thought processes, emotional responses, cognitive functioning, and behavior. Traditionally, mental illnesses can be broadly categorized as non-organic and organic mental diseases. Non-organic psychoses are mainly represented by schizophrenia (SCZ) and major depressive disorder (MDD). Organic mental diseases are caused by brain or somatic diseases, including neurodegenerative diseases and cerebrovascular diseases. The main representative of neurodegenerative diseases is Alzheimer's disease (AD) [9]. Mental disorders exhibit a distinct character, posing a threat to the stability of not just the affected individual, but also their family and the broader society. Previous studies have reported that patients with common mental disorders such as SCZ, MDD, and AD may have a higher risk of falling than healthy people [10–15]. However, an ongoing debate within the medical community persists regarding whether the disease itself or other factors, such as related antipsychotic medication, contribute to an increased risk of falls. In addition, most of the current studies are observational and cannot mitigate the interference of confounding factors and reverse causality.

Mendelian randomization (MR) analysis is a causal inference method that has recently emerged in the field of epidemiology. Genetic variation is congenitally acquired at birth and randomly assigned at conception, an exposure that is usually independent of lifestyle and environmental factors after birth. Based on the core idea of Mendel's second law, MR simulates randomized controlled trials by introducing genetic variation as instrumental variables (IVs) [16]. Theoretically, MR ensures the balance of confounding factors among different groups of genetic variation, thereby minimizing the interference caused by confounding factors and reverse causality. As an innovative statistical method, MR has been extensively utilized to investigate the causal inference between exposure phenotype and disease or outcome, such as the association between coronavirus disease 2019 and subsequent complications, lifestyles and tumors, intestinal flora and disease, as well as cardiac function and frailty index [17–21]. Nonetheless, to date, there have been no studies to identify the causal relationship between mental disorders and risk of falls from the perspective of genetic susceptibility.

Consequently, this study aimed to explore the causal relationship between genetically determined common psychiatric disorders (SCZ, MDD, and AD) and the risk of falls based on univariate, multivariate, and mediating MR.

## Materials and methods

### Study design

**Fig. 1:**
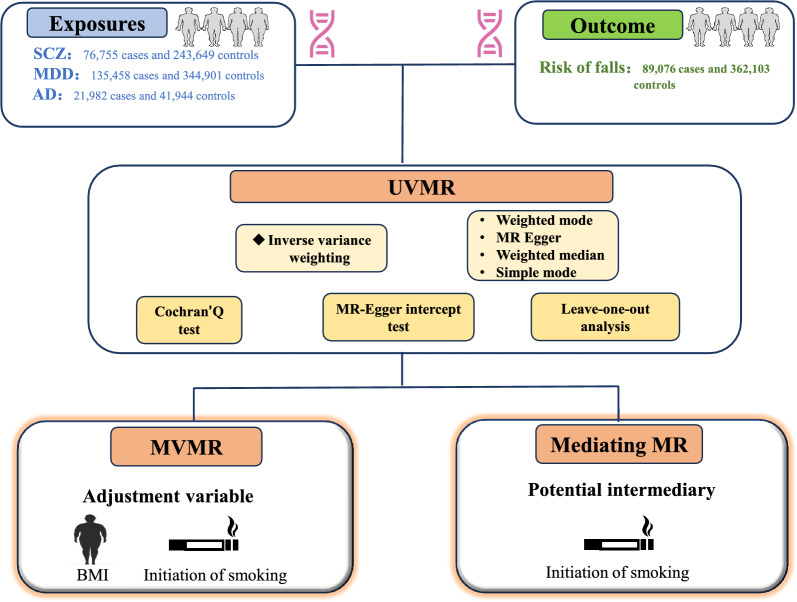
Overview of the study design. SCZ, schizophrenia, MDD, major depressive disorder, AD, Alzheimer's disease, UVMR, Univariate Mendelian randomization, IVW, inverse variance weighting, MVMR, multivariate Mendelian randomization, MR, Mendelian randomization, BMI, Body Mass Index

We obtained data sets of related diseases or phenotypes from the published Genome-Wide Association Research Database (IEU Open GWAS Repository, https://gwas.mrcieu.ac.uk/) to assess the relationship between common psychiatric disorders and the risk of falls. Since the participants in the public database have been approved by the corresponding institutional review committees and ethics committees, no additional ethical approval is required. The overall research design idea is shown in Fig. 1.

### Selection of exposure data sources

This study selected three common psychiatric disorders (SCZ, MDD and AD) as exposures. The GWAS data for SCZ was derived from a large meta-analysis of 320,404 individuals (76,755 cases and 243,649 controls), the largest recently aggregated by Trubetskoy et al. The meta-analysis included 90 core cohorts of European and East Asian ancestry and seven African American and Latino ancestry cohorts from the Psychiatric Genomics Consortium [22]. The GWAS data set for MDD was derived from a meta-analysis by Wray et al., which included 480,359 participants of European descent (135,458 cases and 344,901 controls) [23]. The summary genetic association estimates for AD were obtained from the meta-analysis of GWAS by Kunkle BW et al., which contains individuals of 63,926 European ancestries (21,982 cases and 41,944 controls) [24] (Table 1).

**Table 1:** All data sources used for the MR analysis

Phenotype	Consortium	Years	Samples size	Case Samples size	Population	GWAS ID
*Exposure*						
SCZ	PGC	2022	320,404	76,755	Predominant European (mixed)	ieu-b-5099
MDD	PGC	2018	480,359	135,458	European	ieu-a-1187
AD	ADGC, EADI, CHARGE, GERAD/PERADES	2019	63,926	21,982	European	ieu-b-2
*Outcome*						
Falling risk	GEFOS	2020	451,179	89,076	European	ebi-a-GCST90012857
*Adjustment variable*						
Initiation of smoking Body mass index	GSCAN GIANT	2022 2018	2669 029 681,275	311,629 NA	European European	ieu-b-4877 ieu-b-40

SCZ, schizophrenia; MDD, major depressive disorder; AD, Alzheimer's disease; PGC, Psychiatric Genomics Consortium; ADGC, Alzheimer Disease Genetics Consortium; EADI, European Alzheimer's Disease Initiative; CHARGE, Genetic and Environmental Risk in AD/Defining Genetic, Polygenic and Environmental Risk for Alzheimer’s Disease Consortium; GERAD/PERADES, GEnetic Factors for OSteoporosis Consortium; GSCAN, GWAS and Sequencing Consortium of Alcohol and Nicotine use

### Selection of instrumental variables

The effective IVs should satisfy the following three assumptions: (1) strongly associated with SCZ, MDD, and AD; (2) unrelated to any confounding factors; (3) influenced the risk of falls only through exposure. Hence, prior to MR analysis, strict filtering steps were performed to ensure IVs quality. First, single-nucleotide polymorphisms (SNPs) with *P* < 5E−08 were extracted from GWAS data sets of SCZ, MDD, and AD as potential IVs for genetic susceptibility. Subsequently, the clump data method was used to control that there is no linkage imbalance between the tool variables to acquire independent IVs (defined as *R*^2^ < 0.001, window size = 10,000 kb). In addition, the palindromes SNPs with ambiguity and intermediate allele frequencies were eliminated. The F statistics of each significant SNP were calculated as reported by the formula of the previous research, and the strong tool variables were screened according to the F statistics to avoid the influence of weak IVs [25]. In general, the lower bound for a strong IVs variable is *F* > 10.

### Risk of falls data sources

The risk of falls is the outcome of this study. Pooled statistics on risk of falls were obtained from the latest GWAS analysis conducted by Trajanoska K, including 451,179 participants (89,076 cases and 362,103 controls) of European descent aged 40–69. Cases of falls were defined as participants who gave positive answers to the following questions: “In the last year have you fallen down for any reason (i.e., various extrinsic and intrinsic factors predisposing adults to fall)?” [26] (Table 1).

### Data sources for possible mediators

Previous studies have affirmed significantly correlation between genetically determined body mass index (BMI) and the initiation of smoking (whether an individual had ever smoked regularly) with the risk of falls [27, 28]. BMI and smoking initiation are modifiable factors in daily life. Thus, BMI and smoking initiation were further analyzed as adjustment variables in MVMR and potential mediators of mediating MR. Genetic effects on the BMI were obtained from the Genetic Investigation of Anthropometric Traits consortium, which involves 681,275 participants of European descent. The genetic effect of smoking initiation was derived from the GEnetic Factors for OSteoporosis Consortium, which involved 607,291 subjects of European origin (311,629 cases and 321,173 controls) [7] (Table 1).

### Statistical analysis

In univariate Mendelian randomization (UVMR), a two-sample MR analysis was used to evaluate the direct causal relationship between SCZ, MDD, AD, and risk of falls. Standard inverse variance weighting (IVW) was regarded as the primary analysis method, because it plays a leading role in MR analysis. IVW can obtain a pooled causal estimate by combining the Wald ratio of each SNP with the outcome. Weighted mode, MR Egger, weighted median, and simple mode were utilized to provide more reliable estimates from various aspects to verify the results of IVW. Forest plots and scatter plots were drawn to show the overall trend of results.

The reliability criteria of multivariable Mendelian randomization (MVMR) results include heterogeneity test and horizontal pleiotropy test. Cochran's *Q* tests were performed to identify heterogeneity. Generally speaking, when the *p* value of the Cochran's *Q* test is greater than or equal to 0.05, it indicated no detection heterogeneity in significant SNPs. The absence of horizontal pleiotropy is a crucial assumption of MR. In other words, IVs directly increased the risk of falls only through SCZ, MDD, or AD, not other channels. Therefore, MR–Egger and "leave-one-out" analysis were executed to evaluate the robustness of UVMR results. MR–Egger intercept test can introduce an intercept term to control the influence of invalid tool variables [25]. "Leave-one-out" analysis repeated IVW by discarding each exposure-related SNP to determine whether a single SNP strongly reversed the overall result [25, 29].

After evidence that common psychiatric disorders were significantly associated with the risk of falls, multivariate MR analyses were performed after adjustment for BMI and initiation of smoking to distinguish whether common psychiatric disorders independently increased the risk of falls. Furthermore, we conducted a mediating MR (two-step MR analysis) to identify the mediating effects of BMI and initiation of smoking for further explore the potential mechanism of linking SCZ, MDD, and AD with the risk of falls determined by genetic susceptibility. It should be noted that we only conducted further mediating MR in UVMR-positive associations to estimate the indirect effects of common psychiatric disorders on the risk of falls. Divided the intermediary effect by the total effect, the proportion of the intermediary factor to the total effect was calculated [30].

All data analyses were performed using "Two-Sample MR (version 0.5.6)" and "Mendelian randomization (version 0.5.1)" packages of R (version 4.2.1). *P* < 0.05 was considered a statistically significant difference.

## Results

### Results of UVMR analysis

After strict filtering conditions, 217 SNPs of SCZ, 36 SNPs of MDD, and 21 SNPs of AD were selected as IVs, respectively. The F statistics of all IVs ranged from 29.59 to 962.35, without interference from weak IVs. More features of IVs are shown in Additional file 3: Table S1.

**Fig. 2:**
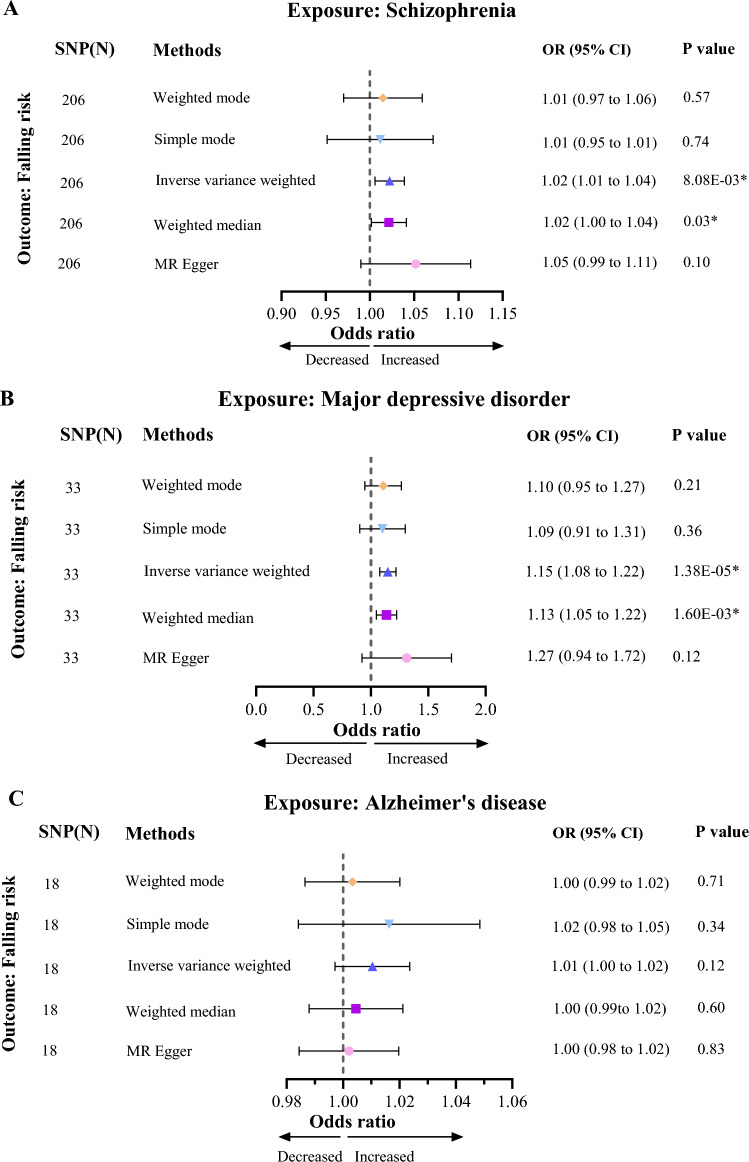
Causal effect of schizophrenia, major depressive disorder and Alzheimer's disease on risk of falls in univariate Mendelian randomization analysis. **A** Causal effect of schizophrenia on risk of falls. **B** Causal effect of major depressive disorder on risk of falls. **C** Causal effect of schizophrenia on risk of falls on risk of falls. CI, confidence interval

In UVMR analysis, the results of the IVW method presented that genetic predisposition to SCZ was significantly associated with an increased risk of falls (OR 1.02, 95% CI 1.01–1.04, *p* = 8.03E−03). Similarly, genetically determined MDD was significantly associated with an increased risk of falls (OR 1.15, 95% CI 1.08–1.22, *p* = 1.38E−05). Astonishingly, there was no significant correlation between genetically determined AD and risk of falls (OR 1.01, 95% CI 1.00–1.02, *p* = 0.12) (Fig. 2). The above results are equally significant in the weighted median method. In addition, the direction of weighted mode, MR Egger, and simple mode is consistent with the results of IVW, albeit not statistically significant (Additional file 1: Fig. S1). The results of Cochran's *Q* test indicated that heterogeneity only the association between genetic predisposition to SCZ and risk of fall. However, the IVW results remained unaffected due to employing a random effects model. The MR–Egger intercept test of the results for three diseases and risk of falls showed that the intercept values were all close to 0 and *P* > 0.05 (Table 2). Therefore, there is no horizontal multiplicity in the results of UVMR. "Leave-one-out" analysis displayed that there was no single anomalous SNP driving the overall result in the opposite direction (Additional file 2: Fig. S2). Accordingly, our results were robust and trustworthy.

**Table 2:** Heterogeneity and pleiotropy analysis of SCZ, MDD, and AD on risk of falls

Data sets	Heterogeneity test			MR–Egger intercept test		
	*Q*	*Q*_*df*	*p* value	intercept	SE	*p* value
SCZ	382.47	204	5.39E−13	− 1.70E−03	1.78E−03	0.34
MDD	44.97	31	0.50	− 3.48E−03	4.95E−03	0.49
AD	18.02	16	0.32	2.72E−03	2.04E−03	0.20

SCZ, schizophrenia, MDD, major depressive disorder, AD, Alzheimer's disease. Q, Cochran Q statistics, SE, standard error. The significance level is defined to be *p* < 0.05

### Results of MVMR analysis

**Fig. 3:**
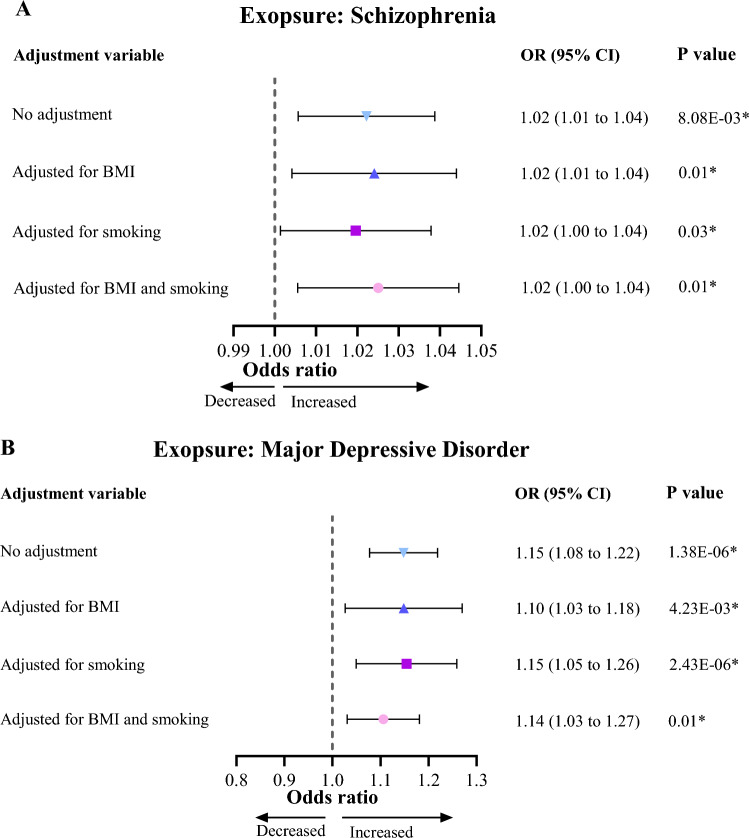
Causal effect of schizophrenia, major depressive disorder on risk of falls in multivariate Mendelian randomization analysis. **A** Causal effect of schizophrenia on risk of falls after adjusting body mass index or/and smoking initiation. **B** Causal effect of major depressive disorder on risk of falls after adjusting body mass index or/and smoking initiation. CI, confidence interval

The results of MVMR analysis, genetic predisposition to SCZ (OR 1.02, 95% CI 1.01–1.04, *p* = 0.01) and MDD (OR 1.10, 95% CI 1.03–1.18, *p* = 4.23E−03) were still significantly associated with an increased risk of falls after adjusting for BMI alone. The causal association between genetic predisposition to SCZ (OR 1.02, 95% CI 1.00–1.04, *p* = 0.03) and MDD (OR 1.15, 95% CI 1.05–1.26, *p* = 2.43E−06) and increased risk of falls remained significant after adjusting for smoking initiation alone. The association between genetic predisposition to SCZ (OR 1.02, 95% CI 1.01–1.04, *p* = 0.01) and MDD (OR 1.14, 95% CI 1.03–1.27, *p* = 0.01) and increased risk of falls remained significant after adjustment for BMI and smoking initiation (Fig. 3).

### Results of mediating MR analysis

**Fig. 4:**
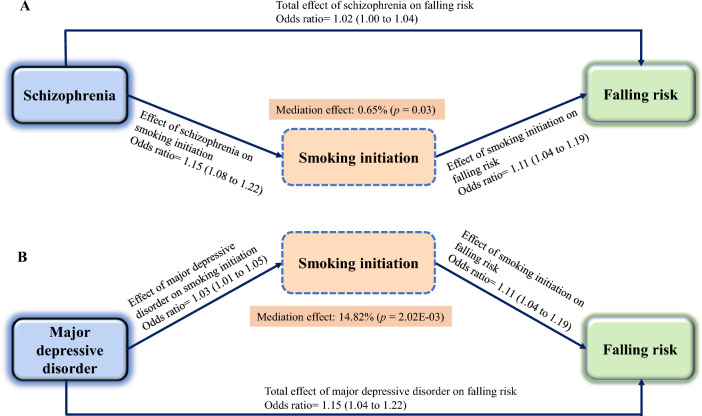
Causal directed acyclic graph showing the effect mediated by smoking initiation for the causal associations of schizophrenia and major depressive disorder on the risk of falls. **A** Effect mediated by smoking initiation for the causal associations of schizophrenia on the risk of falls. **B** Effect mediated by smoking initiation for the causal associations of major depressive disorder on the risk of falls

Mediating MR results showed that smoking initiation mediated 0.65% (*P* = 0.03) of the causal effect of SCZ on the risk of falls. For the causal effects of MDD on the risk of falls, smoking initiation mediated by 14.82% (*P* = 2.02E−03) (Fig. 4). Nevertheless, BMI was not a potential agent for the causal effects of SCZ or MDD on the risk of falls.

## Discussion

We performed UVMR, MVMR, and mediated MR to systematically explore the causal relationship between genetic predisposition to three common psychiatric disorders and the risk of falls. We found that genetically determined SCZ and MDD were significantly associated with an increased risk of falls. MVMR showed that after adjusting for two common interfering factors (BMI and smoking initiation), genetically determined SCZ and MDD were independently associated with increased risk of falls, respectively. Initiation of smoking mediated the partially genetically determined effects of SCZ and MDD on the increased risk of falls.

Several previous epidemiological studies have investigated the association of SCZ, MDD, or AD with the risk of falls. As is commonly recognized, SCZ, MDD, or AD were often combined with other diseases and multiple drug abuse. Given evidence suggested that the increased risk of falls in patients with mental diseases may be attributed to a variety of factors, including psychiatric symptoms, cognitive impairment, side effects of psychotropic medications, and comorbidities [31]. Variations in sample size, geographical location, and study design could also cause the heterogeneity of research results. The strength of MR was that it could effectively avoid the influence of reverse causality and various deviations. In this MR Study, all significant SNPs served as strong IVs. While the biological function of significant SNPs remains unclear, they may provide a basis for subsequent genetic studies. In addition, the data samples of exposure and outcome were not duplicated. The overwhelming majority of the exposed data came from European origin, and the outcome data derived from European ancestry, which avoided the bias caused by different races to some extent. The random effect IVW model was used to eliminate the influence of heterogeneity as far as possible. All the results were free of horizontal pleiotropy. As a consequence, our MR results were reliable and provided more powerful evidence for the causal relationship between SCZ and MD and risk of falls at the genetic level.

A meta-analysis of 7355 patients in 16 studies showed that the total annual fall rate of patients with psychiatric disorders aged 60 and above was 8.74%, and the total lifetime fall rate was 17.25% [8]. According to a study conducted in Taiwan, SCZ patients had a higher risk of recurrent falls after reaching middle age [10]. A prospective cohort study demonstrated that 13.3% of the 120 SCZ patients fell during the 3-month follow-up period, and the current history of falls predicted the future risk of falls [12]. An Australian retrospective study proved that psychotropic substance use was associated with an increased risk of falls in women [32]. Consistent with most traditional observational studies, our UVMR results corroborated that genetic susceptibility to SCZ increases the risk of falls. Furthermore, the results of MVMR supported the findings of UVMR, illustrating that the causal association between SCZ and increased risk of falls was independent of initiation of smoking and BMI. It was speculated that patients with individuals with SCZ may undergo accelerated aging compared to the general elderly population, resulting in poorer physical function and a higher risk of falls.

A cohort study of 12,392 participants from China Health and Retirement Longitudinal Study found that high depressive symptoms were significantly related to the risk of falls and injurious falls [15]. An American meta-analysis of 45,590 subjects corroborated that the presence of depressive symptoms was an autonomous risk factor for falls in the elderly population [33]. Tasha Kvelde et al. confirmed that depressive symptoms increased the risk of falls in the elderly and were independent of the side effects of antidepressants [34]. Consistent with our MR results, genetically determined MDD increased the risk of falls, and this causal relationship was independent of smoking initiation and BMI.

AD patients were reported to be two to three times more likely to fall compared to non-AD patients [35]. A prospective cohort study of 5581 participants showed that AD patients were nearly 15% more likely to have a fall within a year than non-AD patients [36]. Northern Sweden reported that the incidence of falls among patients in a geriatric psychiatric hospital was about 40% within 2 years, and the fall was induced by disease symptoms and drug side effects alone or in combination with other factors [37]. Conversely, our results of MR verified that AD was not causally associated with an increased risk of falls, at least at the genetic level. However, this does not mean we do not need to pay attention to the fall risk of patients with AD. One possible explanation was that the increased risk of falls in patients with AD mainly depended on the potential regulatory effects of other external factors (such as drug side effects and poor mobility). Stark et al. confirmed that the incidence of falls is higher in the pre-clinical AD patients. Colony stimulating factor biomarkers can utilized for predicting the risk of falls in pre-clinical AD patients [38]. Audrey believes that functional activity indicators (gait and balance) may exhibit impairments in pre-clinical AD patients prior to the onset of symptomatic cognitive dysfunction and lead to an increased risk of falls [39]. Hence, although no association was observed between the genetic liability of AD and falls, it may impossible to assess the meaning of this negative result in present study. More data sets were still needed to verify the results further.

Current studies quantify the mediating role of BMI and smoking initiation between genetic susceptibility to SCZ and MDD and the risk of falls. Our findings indicate that smoking initiation plays a slight mediating role in the causal effect of SCZ on the increase of fall risk, while it plays a substantial mediating role in the effect of MDD on the increase of falling risk. The potential reason for the heightened risk of falling associated with smoking was the induced skeletal muscle atrophy. Previous study indicated that smoking initiated interactions between carbon monoxide and hemoglobin, myoglobin, and components of the respiratory chain. This interaction hindered the capacity of mitochondrial oxygen delivery and ATP synthesis, ultimately resulting in a decrease in the endurance of skeletal muscle contractions [40]. Muscle atrophy F-box (MAFBx) expression was heightened in the quadriceps of smoker [41]. In addition, muscle fiber atrophy, reduced muscle mass, and progressive myosin degradation were observed in rodents exposed to cigarette smoke [42–45]. A cross-sectional study involving 7688 elderly Taiwanese males revealed that smoking had a detrimental impact on the cardiopulmonary function, muscular endurance, flexibility, and balance performance of elderly men. Smoking cessation might restore flexibility and static balance performance in the elderly [46]. In other words, the effects of smoking on risk of fall were reversible. Smoking as a habit that can be changed in daily life. Therefore, smoking control may more or less reduce the risk of falls in patients with SCZ and MDD. Previous research has indicated that individuals with SCZ and MDD are more likely to experience reduced physical activity, orthostatic hypotension, type 2 diabetes, and muscle weakness [47–50]. When we attempted to establish mediation models using these factors as potential intermediaries in the relationship between SCZ or MDD and the risk of falls, all results turned out to be null. More effective intermediaries still need to be further explored.

To the best of our knowledge, this is the first study using MR to explore the causal association between SCZ, MDD, AD, and risk the falls from the perspective of genetic susceptibility. Undoubtedly, there were also several limitations in this study. First, we could not further explore the impact of genetically determined SCZ, MDD, and AD on risk of falls after stratification by gender due to data limitations. Second, the GWAS data sets in this study were mainly from people of European origin, and the conclusions of the study could not be hastily applied to populations of other races. Last but not least, the positive outcome of this study relied heavily on self-reported phenotypes, which may have been influenced by subject memory bias or questionnaire quality.

## Conclusions

In a nutshell, our MR Study provides genetic evidence for a causal relationship of individuals with SCZ and MDD on an increased risk of falls, and further research is needed to reveal the potential biological mechanism of this causal relationship. Quitting smoking may be an effective measure to reduce the risk of falls in patients with SCZ and MDD. Although there is no causal relationship between AD and risk of falls at the genetic level, it is still necessary to explore other acquired and external factors to routinely prevent falls.

### Supplementary Information

**Additional file 1: Fig. S1**. Scatter plots to visualize causal effect of SCZ, MDD, and AD on risk of falls in univariate Mendelian randomization analysis. A, scatter plot to causal effect of SCZ on risk of falls, B, scatter plot to causal effect of MDD on risk of falls, C, scatter plot to causal effect of AD on risk of falls. SCZ, schizophrenia, MDD, major depressive disorder, AD, Alzheimer's disease.

**Additional file 2: Fig. S2**. Forest plots to visualize causal effect for each single SNP of SCZ, MDD, and AD on risk of falls in univariate Mendelian randomization analysis. A, forest plots to visualize causal effect for each single SNP of SCZ on risk of falls, B, forest plots to visualize causal effect for each single SNP of MDD on risk of falls, C, forest plots to visualize causal effect for each single SNP of AD on risk of falls. SCZ, schizophrenia, MDD, major depressive disorder, AD, Alzheimer's disease.

**Additional file 3: Table S1**. Instrumental SNPs from GWAS of schizophrenia, major depressive disorder, and Alzheimer’s disease.

## Data Availability

All data generated or analyzed during this study are included in this article and its additional materials.

## Abbreviations

OP: Osteoporosis
SCZ: Schizophrenia
MDD: Major depressive disorder
AD: Alzheimer's disease
GWAS: Genome-wide association studies
IVs: Instrumental variables
CI: Confidence interval
MR: Mendelian randomization
SNP: Single-nucleotide polymorphism
UVMR: Univariate Mendelian randomization
MVMR: Multivariate Mendelian randomization
BMI: Body mass index
