# Supplementary material for: Causal associations between genetically determined common psychiatric disorders and the risk of falls: evidence from Mendelian randomization
Source: Eur J Med Res. 2023 Dec 9;28:578. doi: 10.1186/s40001-023-01502-y (PMC10709873; doi:10.1186/s40001-023-01502-y)
Supplement: Supplementary file 1 — Additional file 1: Fig. S1. Scatter plots to visualize causal effect of SCZ, MDD, and AD on risk of falls in univariate Mendelian randomization analysis. A, scatter plot to causal effect of SCZ on risk of falls, B, scatter plot to causal effect of MDD on risk of falls, C, scatter plot to causal effect of AD on risk of falls. SCZ, schizophrenia, MDD, major depressive disorder, AD, Alzheimer's disease. [file 40001_2023_1502_MOESM1_ESM.docx]

A

B

C

**Supplementary Figure 1.** Scatter plots to visualize causal effect of SCZ, MDD, and AD on risk of falls in univariate Mendelian randomization analysis. A, scatter plot to causal effect of SCZ on risk of falls, B, scatter plot to causal effect of MDD on risk of falls, C, scatter plot to causal effect of MDD on risk of falls. SCZ, schizophrenia, MDD, major depressive disorder, AD, Alzheimer's disease.
