# Supplementary material for: Causal associations between genetically determined common psychiatric disorders and the risk of falls: evidence from Mendelian randomization
Source: Eur J Med Res. 2023 Dec 9;28:578. doi: 10.1186/s40001-023-01502-y (PMC10709873; doi:10.1186/s40001-023-01502-y)
Supplement: Supplementary file 2 — Additional file 2: Fig. S2. Forest plots to visualize causal effect for each single SNP of SCZ, MDD, and AD on risk of falls in univariate Mendelian randomization analysis. A, forest plots to visualize causal effect for each single SNP of SCZ on risk of falls, B, forest plots to visualize causal effect for each single SNP of MDD on risk of falls, C, forest plots to visualize causal effect for each single SNP of AD on risk of falls. SCZ, schizophrenia, MDD, major depressive disorder, AD, Alzheimer's disease. [file 40001_2023_1502_MOESM2_ESM.docx]

A

B

C

**Supplementary Figure 2.** Forest plots to visualize causal effect for each single SNP of SCZ, MDD, and AD on risk of falls in univariate Mendelian randomization analysis. A, forest plots to visualize causal effect for each single SNP of SCZ on risk of falls, B, forest plots to visualize causal effect for each single SNP of MDD on risk of falls, C, forest plots to visualize causal effect for each single SNP of AD on risk of falls. SCZ, schizophrenia, MDD, major depressive disorder, AD, Alzheimer's disease.
